# Complete Genome Sequence of a CTX-M-15-Producing *Escherichia coli* Strain from the *H*30Rx Subclone of Sequence Type 131 from a Patient with Recurrent Urinary Tract Infections, Closely Related to a Lethal Urosepsis Isolate from the Patient’s Sister

**DOI:** 10.1128/genomeA.00334-16

**Published:** 2016-05-12

**Authors:** Timothy J. Johnson, Maliha Aziz, Cindy M. Liu, Evgeni Sokurenko, Dagmara I. Kisiela, Sandip Paul, Paal Andersen, James R. Johnson, Lance B. Price

**Affiliations:** a Department of Veterinary and Biomedical Sciences, College of Veterinary Medicine, University of Minnesota, St. Paul, Minnesota, USA; b Department of Environmental and Occupational Health, George Washington University, Washington, District of Columbia, USA; c Division of Pathogen Genomics, Translational Genomics Research Institute (TGen), Flagstaff, Arizona, USA; d Department of Microbiology, University of Washington School of Medicine, Seattle, Washington, USA; e Microbiology and Infection Control, Statens Serum Institut, Copenhagen, Denmark; f Veterans Affairs Medical Center and University of Minnesota, Minneapolis, Minnesota, USA

## Abstract

We report here the complete genome sequence, including five plasmid sequences, of *Escherichia coli* sequence type 131 (ST131) strain JJ1887. The strain was isolated in 2007 in the United States from a patient with recurrent cystitis, whose caregiver sister died from urosepsis caused by a nearly identical strain.

## GENOME ANNOUNCEMENT

*Escherichia coli* sequence type 131 (ST131) is currently the most prevalent extraintestinal *E. coli* lineage (1). Its ST131-*H*30 sublineage accounts for most *E. coli* isolates that are fluoroquinolone resistant and produce extended-spectrum β-lactamases (ESBLs) (2). Carriage of *bla*_CTX-M-15_ is associated with the ST131-*H*30Rx sublineage within ST131-*H*30 (3). ST131-*H*30 is associated with recurrent urinary tract infections, pyelonephritis, and urosepsis (1, 4–7). We sequenced two *E. coli* clinical isolates from two adult sisters, one with recurrent *E. coli* cystitis, and the other with fatal *E. coli* urosepsis that developed after caring for her sister. We previously reported the complete genome sequence of the deceased sister’s fatal *E. coli* strain (JJ1886), from the ST131-*H*30Rx sublineage (8). Here, we present the complete genome sequence of the surviving sister’s *E. coli* strain (JJ1887), which is also from ST131-*H*30Rx and nearly identical to JJ1886. Whether the sisters’ distinct clinical outcomes are attributable to host response differences, strain-specific features, or other factors, the JJ1887 genome represents a valuable added resource for understanding the pathogenicity of the ST131-*H*30Rx lineage.

JJ1887 was sequenced on the PacBio platform (P5-C3 chemistry), which generated 66,442 raw PacBio reads (mean read length, 10,942 bp; total nucleotides, 764,654,567). The PacBio sequence reads were manually error corrected using Illumina short reads (125× coverage, TruSeq chemistry) using CLC Genomics Workbench version 7. The resultant data were assembled using the Hierarchical Genome Assembly Process (HGAP) version 3 in the PacBio single-molecule real-time (SMRT*)* Portal, which produced a circular chromosome and five closed plasmids, with a mean coverage of 118×. The short-read mapping was further analyzed with Delly (9) and the Northern Arizona SNP Pipeline (NASP) (http://TGenNorth.github.io/NASP). The verified sequences were annotated using Prokka 1.10 (10) and the NCBI Prokaryotic Genome Annotation Pipeline (PGAP). Antimicrobial resistance genes were identified using ResFinder 2.1 (11), and phage regions were identified using PHAST (12).

The complete genome of the JJ1887 chromosome comprises a 5,081,061-bp chromosome with a G+C content of 50.78%. It includes 4,773 coding sequences (CDSs), 89 tRNAs, 22 rRNA features, and 7 intact prophage regions, one of which (57.4 kb) harbors *bla*_CTX-M-15_.

JJ1887 contains five plasmids, of which pJJ1887-1, -2, and -3 are identical to plasmids in JJ1886 (8). According to PlasmidFinder 1.3 (13), pJJ1887-1 (1,552 bp) is a Col(MG828) plasmid with two CDSs; plasmid pJJ1887-2 (5,167 bp) is a Col156 plasmid with five CDSs; pJJ1887-3 (5,631 bp) is a ColE1-like plasmid with five CDSs; pJJ1887-4 (107,507 bp) has both RepFIA and RepFII replicons, with 125 CDSs; and pJJ1887-5 (130,603 bp) has both RepFIB and RepFII replicons, with 158 CDSs. *bla*_TEM-1B_, *bla*_OXA-1_ in pJJ1887-4 and *bla*_TEM-1B_ in pJJ1887-5 confer resistance to β-lactams, and the *aac(6′)-Ib-cr* gene in pJJ1887-4 codes for fluoroquinolone resistance. pJJ1887-5 also carries genes conferring resistance to aminoglycosides [*aac-(3)-IIa*], macrolides [*mph*(*A*)], tetracyclines [*tet*(*B*)], trimethoprim (*dfrB4*), sulfonamides (*sul1*), and fluoroquinolones (*qepA*).

### Nucleotide sequence accession numbers.

The complete sequences of the chromosome and plasmids of *E. coli* JJ1887 have been deposited in GenBank under accession numbers CP014316 to CP014321.
